# Economic and Cultural Assessment of the DASH Eating Plan for Low-Income African Americans: An Integrative Review

**DOI:** 10.3390/ijerph21111480

**Published:** 2024-11-07

**Authors:** Brandi M. White, Kendra OoNorasak, Nadia A. Sesay, Deidra Haskins, Cayla M. Robinson

**Affiliations:** College of Health Sciences, University of Kentucky, 900 South Limestone Street, Room 209C, Lexington, KY 40536-0200, USA; kendra.oonorasak@uky.edu (K.O.); nadia.sesay@uky.edu (N.A.S.); diedra.haskins@uky.edu (D.H.); cayla.robinson17@uky.edu (C.M.R.)

**Keywords:** DASH diet, African Americans, low-income populations, hypertension, food literacy

## Abstract

Diet is one modifiable risk factor for hypertension. The low-sodium DASH (Dietary Approaches to Stop Hypertension) eating plan has been shown to significantly reduce the risk of hypertension and cardiovascular disease. However, there is a lack of available health information on the economic feasibility and cultural acceptability of DASH for low-income African American (AA) populations who are at the most risk for hypertension. An integrative review was conducted to summarize empirical literature on the economic feasibility and cultural acceptability of the DASH plan for low-income AAs using these databases: PubMed, EMBASE, CINAHL Complete, AGRICOLA, Web of Science Core Collection, ProQuest’s Dissertations, Theses Citation Index, and Google Scholar. Study elements from articles in the final analysis were extracted. Eleven (11) published works met the study’s inclusion criteria. Major themes were the availability and access of healthy foods, economic impact of obtaining healthy foods, material resources for cooking, food literacy, and the cultural acceptability of the DASH plan. These findings suggest that cost and cultural familiarity inhibit low-income AAs from benefiting from the DASH plan. Additional research is needed to develop and pilot test low-cost, culturally sensitive DASH eating plans for low-income AAs.

## 1. Introduction

Hypertension affects almost half of American adults 20 years and older (47.3%) [1]. Despite efforts to reduce hypertension rates in the United States, non-Hispanic Black/African Americans continue to have higher rates compared to their non-Hispanic White, non-Hispanic Asian, and Hispanic counterparts [1]. Further, African Americans reportedly have the highest rates of hypertension in the world [1]. African Americans also reportedly have poorer blood pressure control [2,3]. To reduce hypertension disparities, there is an urgent need for effective treatments to improve blood pressure control and effectively treat hypertension for African American populations.

A heart-healthy diet is important for the prevention and control of hypertension [4,5]. In 2019, the American Heart Association (AHA) and the American College of Cardiology released guidelines for the primary prevention of hypertension that emphasized healthy lifestyle changes, such as a heart-healthy eating plan, to promote cardiovascular health via a holistic approach instead of pharmacologic therapy [6]. One such eating plan that is highly effective at improving blood pressure control and reducing hypertension risk is the low-sodium DASH (Dietary Approaches to Stop Hypertension) [7,8]. The evidence-based DASH eating plan promotes eating fruits/vegetables, whole grains, fat-free/low-fat dairy products, poultry, fish, beans, nuts, and vegetable oils; limited foods high in saturated fat; and limited daily consumption of sodium, at 1500–2300 milligrams (mg), and sugar [9]. A 2023 study found that DASH has a more pronounced positive effect on reducing cardiovascular disease risk for African Americans compared to Whites [10]. Because of the low-sodium DASH’s effectiveness, it is a viable dietary strategy to reduce hypertension disparities, particularly among African Americans.

Yet African Americans are reportedly less likely to adhere to the low-sodium DASH [11], emphasizing the importance of culturally responsive DASH recommendations for African Americans. For low-income African Americans and other low-income groups, the low-sodium DASH adherence may be more challenging because of financial barriers that limit healthy food options. A 2012 review conducted by Spencer and colleagues identified several barriers to DASH adherence for African American women, including the low availability of DASH-recommended foods in low-income African American communities, the high cost of healthy foods, and the lack of culturally tailored DASH plans (e.g., soul food) [12]. These findings suggest the need for low-cost and low-sodium DASH recipes and plans that are tailored to African American food traditions. 

This study updates the Spencer review [12] by conducting an integrative review to summarize empirical literature on the economic feasibility and cultural acceptability of the low-sodium DASH eating plan for low-income African Americans. An integrative review provides a summary and critique of a wider range of empirical evidence compared to other types of literature reviews [13]. It also permits the inclusion of diverse methodologies, such as experimental and non-experimental research, which may provide more useful and usable information to inform evidence-based practice [13]. These findings can provide guidance to healthcare professionals and public health practitioners on how to provide culturally sensitive heart-healthy dietary recommendations to low-income African American patient populations and communities.

## 2. Materials and Methods

### 2.1. Eligibility Criteria

Inclusion and exclusion criteria are listed in the registered protocol, but also provided here [14]. Inclusion criteria: (1) reports information about adherence to the low-sodium DASH eating plan; (2) adult populations with or without a hypertension diagnosis; (3) observational or interventional studies, qualitative or mixed-methods studies, process or outcome evaluations, descriptive research, dissertations, or conference proceedings; (4) published on or after 1 January 1997 to 26 January 2024 (efficacy the DASH diet was established in 1997) [15]; (5) published in the English language; (6) and research conducted in the United States. Exclusion criteria: (1) studies that do not report findings on low-income African American women; (2) studies on immigrant populations; (3) and literature reviews, systematic reviews and clinical trial registries, study protocols, editorials, blogs, or commentary papers.

### 2.2. Search Strategy

A health sciences librarian, in conjunction with the research team, devised a systematic search which focused on Black Americans and the DASH plan. The librarian adhered to the PRISMA-Searching extension [16]. The search was originally constructed in PubMed (Pubmed.gov) and then translated to 6 additional databases. Those databases are Elsevier’s EMBASE, EBSCOhost’s CINAHL Complete and Agricola, the Web of Science Core Collection and Web of Science Dissertations & Theses ProQuest index through Clarivate, and finally Google Scholar. Full database coverages and indices can be found via the registered protocol in Open Science Framework [14]. A date limit of 1997 was used due to the DASH diet not being created until 1997 [15].

Searches were translated to the additional databases using the Systematic Review Accelerator’s Polyglot tool [17]. Results were exported to EndNote version 21 for deduplication on 23 February 2024. An initial deduplication was conducted in EndNote, and then a supplementary deduplication was conducted by hand using the Systematic Review Accelerator’s Deduplicator tool [17]. Full search strategies are hosted in the SearchRXIV search strategy repository [18]. This ensures reproducibility of the search. 

**Figure 1:**
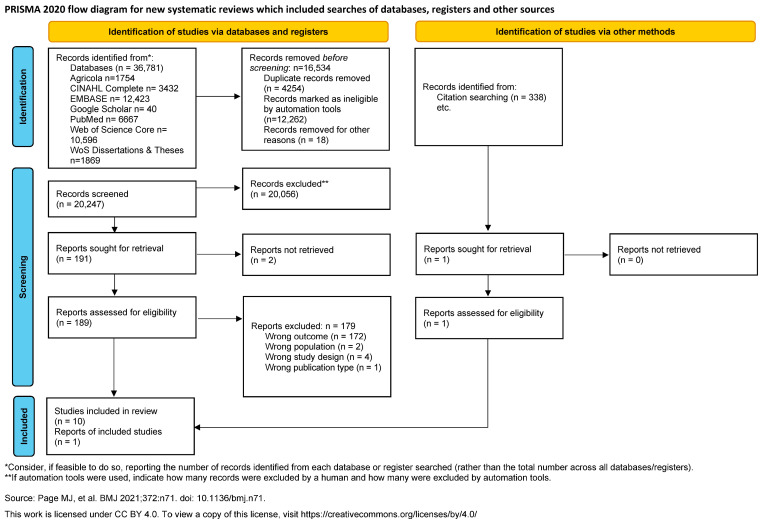
PRISMA flow diagram [21].

Upon the completion of both deduplication steps, the librarian exported the results into the Rayyan systematic review software [19]. Independent and masked title and abstract and full-text screening were conducted using Rayyan by two researchers on the team. Conflicts were discussed by the reviewers and handled using Rayyan. Finally, once the articles were selected for final inclusion, the medical librarian conducted citation searching using the SpiderCite tool from the Systematic Review Accelerator [20]. These articles were then hand-searched, and three of them were conference abstracts and thus did not have cited or citing articles. Figure 1 provides the PRISMA flow diagram for the integrative review. 

### 2.3. Data Extraction

Two reviewers extracted key elements from articles included in the final review. Extractions included study purpose, study design, methods, inclusion/exclusion criteria, sample size, patient characteristics and qualitative findings related to the economic feasibility and cultural acceptability of the DASH eating plan for low-income African Americans (Table 1 and Table 2).

### 2.4. Data Analysis 

To analyze qualitative findings, thematic analysis was used. Thematic analysis allows for the identification, organization, and interpretation of patterns or themes within qualitative data, including integrative reviews [33]. Data was coded using inductive approaches. Major themes and subthemes were identified, defined, and agreed upon by members of the research.

## 3. Results

### 3.1. Characteristics of Included Studies

Bertoni et al. explored the perceptions of DASH and the food environment of African Americans. Participants had high blood pressure but were taking fewer than three antihypertensive medications [22]. The mixed-methods study employed focus groups and a quantitative food environmental assessment. Participants aged 21 years and older were recruited from two zip codes with high percentages of low-income African American residents [22]. Targeting an older population, Jones et al. conducted focus groups with African Americans aged 60 and older living in senior housing to document challenges with dietary hypertension self-management through DASH [23]. Wright et al. also examined the feasibility and acceptability of DASH intervention among older community-dwelling adults (M_age_ = 74; SD = 5.9) [26]. Additional inclusion criteria were hypertension diagnosis and mild cognitive impairment, indicated by a questionnaire [26].

As mentioned, African Americans are less likely to adhere to DASH despite evidence of the eating plan’s effectiveness. Included studies examined reasons for lagging adoption of DASH among this population. Brown et al. conducted qualitative interviews with African Americans with hypertension and chronic kidney disease (CKD) enrolled in a dietary intervention trial to determine barriers and facilitators to DASH adherence [29]. A conference abstract and research article examined DASH reception among African Americans with hypertension and CKD in one study [31,32]. Tyson et al. [31,32] assessed DASH perceptions and identified barriers and facilitators to DASH adherence among African American adults with CKD stage 3 or 4. Additional participant characteristics reported in Tyson were full-time work status (27%) and annual household income <$25,000 (39%) [32]. Slightly more than half of participants (52%) reported rarely/never having enough money left over to purchase healthy food in a typical month [32].

One reported barrier to DASH adoption among African Americans is the low cultural relevance of DASH-recommended foods. Rankins et al. modified traditional soul food into a DASH eating plan and conducted a pilot test of the modified DASH plan with low-income African American women [25]. Karanja et al. assessed the acceptability of DASH among adults with prehypertension and stage 1 hypertension [24]. Using a randomized crossover trial, they conducted a subgroup analysis by race to examine acceptability of DASH among participants in an outpatient feeding trial [24]. Crews et al. examined whether availability of kitchenware is another barrier to this DASH eating plan [30]. Specifically, Crews and colleagues conducted qualitative interviews and home inspections to determine whether presence of DASH-accordant foods and kitchen appliances needed to prepare DASH meals among urban African Americans with uncontrolled hypertension enrolled in a randomized control trial [30].

Vollmer et al. designed a randomized feeding trial and cross-sectional analysis to compare barriers and facilitators to DASH adherence among African American and non-Hispanic White participants [27]. Among African American participants (*n* = 275), most were female (59%) and had 1–4 years college education (64%). Nearly half (45%) of African American females had household incomes less than $30,000 [27]. Young et al. also performed a DASH comparison between two populations [28]. Specifically, they examined barriers to DASH adherence among patients in high- and low-SES communities [28]. Participants surveyed at a high-SES clinic were more likely to be young, female, live in high-SES communities, have higher levels of education and greater household income, and were less likely to be minorities. Participants at the low-SES clinic were more likely to live in low-SES communities and 74% were African American [28].

### 3.2. Major Themes of Published Works in the Review

There were five major themes identified in the studies included in the review: (1) availability and access of healthy foods; (2) the economic cost of healthy foods; (3) material resources for cooking; (4) food literacy; and (5) the cultural acceptability of the DASH eating plan for African Americans. 

#### 3.2.1. Theme 1: Availability and Access of Healthy Foods

This theme referred to the availability and visibility of healthy food options that are DASH-friendly (e.g., fruits and vegetables) within a given environment, such as a grocery store, market, or community. There were conflicting views on whether DASH-friendly healthy foods were available to purchase in low-income African American communities. Four studies reported that the poor availability of healthy foods was a barrier to following a DASH eating plan [22,23,28,29]. One study reported that there was a concern that the availability of fruits, vegetables, and lean meats was low among community participants [22]. Another study found that high-income communities offered 75% of DASH-friendly foods compared to 46% in low-income communities [28]. For those who relied on food banks for groceries, there were reportedly limited healthy food options [23]. Transportation was also identified as a barrier to DASH adherence for those lacking access [29]. On the contrary, qualitative findings from African American adults with chronic kidney disease (CKD) reported that DASH-friendly foods were available and accessible among study participants [31,32]. 

#### 3.2.2. Theme 2: Economic Cost of Healthy Foods

Another major theme was related to the economic cost of healthy foods. This refers to the price consumers must pay to purchase healthy food items that are DASH-friendly, affordability relative to other less healthy options, and cost-control measures. Six studies reported that the high cost of healthy food (e.g., vegetables) was a concern and barrier to DASH adherence [22,23,25,28,29,32]. One study found that living in poverty (<$30,000 annual income) was associated with the lower odds of having whole grains, an important component of the DASH eating plan, among African Americans living in an urban community [30]. The abundance of low-cost high-sodium unhealthy food options (e.g., fast food restaurants) in low-income African American communities was also identified as an economic barrier to DASH adherence [22]. Two studies reported concerns about the spoilage of fresh produce as an economic barrier, indicating that spoiled food would result in higher food costs [22,32]. However, despite these economic concerns, one study reported that healthy eating was a priority for participants and could be accomplished with cost-saving strategies, such as buying low-sodium canned or frozen goods and using discount coupons [32].

#### 3.2.3. Theme 3: Material Resources for Cooking

The third major theme was related to material resources for cooking or the physical tools and equipment necessary to prepare meals (e.g., kitchen appliances). Two studies reported on inadequate cooking equipment and supplies in low-income African American households [30,32]; one of them found that not owning the proper cooking equipment was a barrier to DASH adherence [32]. One study found that low literacy (less than a third-grade level) was associated with the lower odds of having an oven and full-sized refrigerator among urban African Americans with uncontrolled hypertension [30]. 

#### 3.2.4. Theme 4: Food Literacy

The fourth major theme was focused on food literacy. Food literacy consists of the knowledge, skills, and attitudes necessary to make informed decisions about food that contribute to health and well-being (e.g., cooking skills and food preparation techniques) [34]. One study reported as a conference abstract and research article found that inadequate cooking skills were a barrier to DASH adherence among African Americans with CKD [31,32]. In this study of African Americans with CKD who were presented with information about the DASH eating plan, participants found limiting sweets, limiting fats/oils, and meeting daily whole grain targets to be the most challenging [32]. A few participants in this same study reported that the DASH eating plan did not account for African American cultural traditions [32]. The lack of familiarity with measuring serving sizes, partly due to limited food literacy, was noted [32]. Similarly, one study found that DASH recipes used preparation techniques that were not common in the African American community [22]. The authors concluded the need to have easy-to-follow DASH recipes with simple ingredients, and additional education on how to prepare new or unfamiliar foods [32].

#### 3.2.5. Theme 5: Cultural Acceptability of the DASH Eating Plan

The last major theme was the cultural acceptability of the DASH eating plan. This refers to how well the DASH eating plan aligns with and is accepted within African American cultural food practices, preferences, and traditions. Findings on the cultural acceptability of the DASH eating plan were mixed. Three studies reported that the DASH eating plan was not culturally relevant [22,27,32]. The lack of familiarity with DASH foods and recipes were reported as a barrier to DASH adherence in two studies [22,32]. Another study reported that, among African American participants in a controlled DASH feeding trial, 19% disliked DASH friendly foods and 13% believed those foods lacked variety [27]. 

On the other hand, four studies reported that the DASH eating plan was culturally acceptable [24,25,26,31,32]. In a study of community-dwelling older African Americans, participants reported that the DASH intervention was culturally acceptable [26]. Another study found no racial differences between African Americans and non-African Americans in the likability of the DASH eating plan and in the acceptability of main food groups serving sizes (e.g., fruits and vegetables, low-fat dairy products) [24]. In the study of African American adults with CKD, most participants felt the eating plan was culturally compatible, especially because many DASH foods are found in a typical African American diet [31,32]. Participants also noted that the African American diet is not uniform [31,32]. Two studies reported participants being open to finding healthier options to preparing traditional African American foods (e.g., traditional southern or soul food) [25,32]. This included using less pork, salt and sugar, and avoiding fried foods [32]. One study also reported participants stating that eating healthily was more important than following cultural traditions [32].

## 4. Discussion

This integrative review provides an update to the review conducted by Spencer and colleagues [12] by identifying nine additional articles and abstracts that evaluate the economic feasibility and cultural acceptability of the low-sodium DASH plan for low-income African Americans. Several studies reported that healthy foods were not available and accessible for low-income African American populations [22,23,29]; however, one study reported the contrary [31,32]. In addition, the high cost of healthy foods and spoilage concerns regarding fresh produce were barriers to DASH adherence [22,23,25,28,29]. Inadequate cooking equipment/supplies [30,32] and food illiteracy [31,32] were also identified as barriers to DASH adherence. There were mixed findings regarding the cultural acceptability of DASH. Some studies reported that DASH used unfamiliar foods, recipes, and cooking methods without accounting for African American cultural traditions [22,27,32]. In contrast, some studies reported that DASH was culturally acceptable and used foods typically found in traditional African American foods [24,25,26,31,32]. Some participants were also open to modifying traditional African America foods/recipes into healthier options [25,32].

A major finding from this study was the poor availability and accessibility of DASH-friendly foods in low-income African American communities, including fruits, vegetables, and lean meats [22,23], despite Tyson’s study reporting healthy foods being accessible and available [31,32]. However, differences in perceptions about the availability and accessibility of healthy foods between this study and others may have been due to the study sample having a proportion of participants who were not economically disadvantaged (39% annual household income <$25,000) [32]. Moreover, previous studies have found that low-income African American communities, have poor access to low-sodium DASH-friendly foods [35,36,37]. In an audit of grocery stores and fast-food restaurants in St. Louis, MO, low-income African American, White, and mixed-race communities had less access to healthy foods compared to high-income White communities [35]. Similarly, a cross-sectional survey of low-income African American adults in an urban district found that the most common food sources were corner stores that are often stocked with processed, high-calorie and -sodium foods, with very little fresh fruits, vegetables, and whole grains [37].

Structural racism may be a contributing factor to the poor availability and accessibility of healthy foods (i.e., low-sodium DASH-friendly) in low-income African American communities [38]. Structural racism is a “form of racism that is pervasively and deeply embedded in and throughout systems, laws, written or unwritten policies, entrenched practices, and established beliefs and attitudes that produce, condone, and perpetuate widespread unfair treatment of people of color” [39,40]. This includes historical practices like redlining that have led to systemic disinvestments in predominantly African American communities, resulting in fewer grocery stores and supermarkets, and racially segregated communities. Targeted community-level interventions have been shown to increase access and availability of healthy foods in low-income African American communities [41,42,43]. Additional research is needed to understand how to eliminate and repair the effects of structural racism through targeted and effective policies/interventions.

Another major finding of the review was that the high cost of healthy foods (i.e., low-sodium DASH-friendly foods) was a barrier to DASH adherence among low-income African Americans [22,23,25,28,29,32]. Previous studies have reported that cost was a barrier to buying healthy foods among low-income populations [44,45,46]. In a focus group study of low-income adults, high cost was a barrier to buying fresh fruits and vegetables, a core DASH component [44]. The cost of healthy foods was also a barrier to eating healthily among a sample of food pantry participants [45]. DASH-friendly foods, such as fresh fruits, vegetables, and whole grains, are often more expensive than processed, sodium-rich, calorie-dense, and nutrient-poor foods. This price difference makes it difficult for low-income households to prioritize healthier options. To lower healthy food costs, policy- and community-level interventions are needed to strengthen local food systems and increase food-purchasing power. For example, there is evidence that increasing SNAP (Supplemental Nutrition Assistance Program) benefits or incentivizing beneficiaries to buy healthy foods is an effective strategy [47,48]. Additional research is needed to better understand how income, cost of DASH-friendly foods, and race/ethnicity influence individuals’ purchasing power and food decision-making processes.

This review also found that inadequate material resources for cooking, such as kitchen appliances, were a barrier to DASH adherence [30,32]. This may indicate that, without basic cooking supplies, low-income individuals may struggle to prepare meals at home. This can lead to reliance on low-cost pre-packaged or fast foods, which are often high in sodium/sugars and unhealthy fats. In a study of low-income caregivers of young children, having fewer cooking supplies was associated with food insecurity [49]. According to the US Department of Agriculture (USDA), food insecurity is regarded as one’s state of having limited reliable access to and utilization of sufficient, affordable, nutritionally-balanced foods to sustain healthy, active lifestyles, due to the lack of monetary and other resources [50]. Another study among low-income adults found that a lack of cooking supplies contributed to unhealthy eating patterns [51]. A lack of cooking supplies can present challenges to DASH adherence by restricting the ability to control the amount of salt, sugar, and unhealthy fats in meals. Future research and public health programs targeting low-income individuals should first gather information on the availability of and access to necessary resources, including basic cookware and kitchenware, and ask for any effective strategies that the target audience may suggest, ensuring that health promotion program plans are derived from the actual voices and lived experiences of the target audience.

Another major finding in this review was that food illiteracy, or the knowledge, skills, and attitudes necessary to make informed decisions about food that contribute to health and well-being [34], was a barrier to DASH adherence [31,32]. Economic disadvantage may be a driving factor of food illiteracy. There are limited studies on food illiteracy among low-income populations in the United States. However, in a study of low-income adults, health illiteracy was associated with unhealthy cooking preparation techniques [52]. Inadequate access to quality educational opportunities may improve food illiteracy issues among low-income populations. Low-income communities often have underfunded schools with fewer resources, which may include health and nutrition education programs. Without early exposure to food literacy education formally or informally, individuals may grow up without foundational knowledge about life skills like cooking and important tools for basic nutrition and healthy eating. Further, many low-income African American communities are food deserts, areas with limited access to grocery stores that sell fresh, healthy foods [53,54]. This lack of exposure to a variety of healthy foods, i.e., low-sodium DASH-friendly foods, can hinder the development of food literacy. Future research/practice prioritizing food literacy education from preschool age through young adulthood, to expose communities to diverse foods, equip them with tools and resources, and improve their self-confidence in food-related life skills and activities, are warranted to lay down an equitable foundation for the future.

The final major finding in this review were the conflicting results about the cultural acceptability of the low-sodium DASH plan. Culture and ethnicity are important factors in food choice and eating habits for low-income African Americans, as reported in a study in this review [28]. Three studies reported that the DASH plan was not culturally acceptable because foods/recipes did not account for African American food traditions and preparation techniques [22,27,32]. This conflict may be due to perceptions about African American food traditions and preparation techniques not being “healthy.” In a study that explored diet and culture among African Americans, participants perceived eating traditional African American meals, such as soul food, conflicted with a heart-healthy diet; however, adhering to African American food traditions was a way for them to stay connected to their heritage and culture, indicating the importance of accounting for food culture in dietary recommendations [55].

Many African American food traditions are tied to cultural identity and have significant meaning [56]. For many African Americans, these food traditions are a significant part of cultural celebrations (e.g., Juneteenth, Thanksgiving, Christmas, Kwanzaa) and family traditions. Unfortunately, many traditional African American dishes, including soul food, can be high in salt and do not align with a heart-healthy eating plan, such as DASH. Foods like fried chicken, collard greens cooked with pork, sweet potatoes, and macaroni and cheese are staples in many African American households, in addition to unhealthy cooking techniques such as deep frying and cooking with fat/lard. Rejecting these food cultural traditions and replacing them with “healthier alternatives” might be viewed as dismissal of their own cultural identity. One key consideration is how public health and healthcare professionals approach such sensitive, deeply rooted cultural subjects around food, navigate through many barriers, and communicate openly and transparently for better health and wellbeing.

On the other hand, five studies reported that DASH was culturally acceptable, including an emphasis that African American food traditions (i.e., soul food) already use DASH-friendly dishes [24,25,26,31,32]. While African American food traditions are often perceived as unhealthy because they are associated with high-fat/sodium dishes, many soul food dishes include nutrient-rich, DASH-friendly vegetables such as collard/mustard/turnip greens, okra, cabbage, and green beans. In addition, soul food incorporates black-eyed peas, lima beans, and other legumes, and sweet potatoes and yams, all DASH-friendly foods. Despite having DASH-friendly food options, the use of traditional cooking techniques (e.g., deep frying) presents challenges to meeting DASH nutrient guidelines.

Fortunately, two studies in the review reported that African American participants were open to finding healthier options to traditional cooking techniques and ingredients [25,32]. A study included in the review discussed baking or broiling foods instead of frying them, and substituting turkey and chicken for red meats [25]. These findings indicate that African American food traditions such as soul food can be modified into a DASH eating plan and be culturally acceptable. This can be accomplished by emphasizing culturally-preferred vegetables, fruit, and legumes/beans, modified cooking techniques, and reduced sodium using herbs and spices, maintaining the flavor and cultural importance of food traditions while supporting cardiovascular health. Future research/programming should take cultural preferences and traditions into consideration when gathering information on target audience and planning any health promotion activities, while understanding that representing and understanding everyone’s culture and traditions is a challenging task. However, unquestionably, it is a critical factor that every professional and community member should strive towards dismantling racial and cultural segregations still occurring in this society.

Though this integrative review was not driven by any theoretical or conceptual framework, key findings represent many components of food and nutrition insecurity [57]. Key features of food and nutrition security are: (1) access, including physical, economic and sociocultural access; (2) availability of food in one’s environment outside of the household level; (3) effective utilization of proper knowledge, skills, and resources; and (4) global stability [57]. The framework further explains that adequate availability of food alone does not guarantee substantial reliable food access, while satisfactory food access and availability in one’s environment does not assure individuals’ proper utilization of food, especially if they have limited food literacy [57]. As it relates to DASH adherence, food and nutrition security provide the foundation for effective disease management by ensuring that individuals have the resources to maintain a diet that supports heart health and overall well-being. Developing and implementing policies and interventions to improve food and nutrition security are needed to improve access to healthy foods, such as low-sodium DASH-friendly foods [58].

### Study Limitations

This study is not without limitations. First, the findings may be subject to selection bias because studies with significant or positive results are more likely to be published and included. Second, for self-report studies (e.g., qualitative mixed-methods studies), social desirability and response bias may skew findings. Third, studies may have included African American populations who had higher incomes, thus not accurately reflecting economic barriers to DASH adherence. Lastly, results may not be generalizable to all low-income African American populations.

## 5. Conclusions

These findings highlight the need to address the economic and cultural barriers to low-sodium DASH adherence among low-income African Americans, a population that bears a heavier burden of hypertension compared to their counterparts. The limited availability and accessibility of low-cost and -sodium DASH-friendly foods are major challenges to DASH adherence. Policies and programs that increase healthy food purchasing power (e.g., increased SNAP beneficiaries, subsidies) and strengthen sustainable local food systems can help with alleviating economic barriers. In addition, the cultural acceptability of DASH and other heart-healthy eating plans must be addressed to remove barriers to adherence. Promoting the use of traditional African American foods that are DASH friendly, and modifying cooking techniques and ingredients into healthier alternatives can increase adherence to low-sodium DASH. To address economic and cultural barriers to DASH adherence, policymakers, healthcare providers, and communities must work together to make heart-healthy choices, such as the DASH eating plan, which is more accessible, affordable, and aligned with cultural preferences. Further, food and nutrition security must become a priority. In this way, health equity can be advanced by reducing the burden of hypertension in low-income African American communities.

## Figures and Tables

**Table 1:** Overview of the studies included in the final analysis (*n* = 11).

Article Title	First Author (Year)	Study Objective	Study Design	Methods	Inclusion Criteria	Exclusion Criteria	Sample Size	Participant Characteristics
A multilevel assessment of barriers to adoption of DASH among African Americans of low socioeconomic status	Bertoni (2011) [22]	To examine perceptions of DASH; To assess food environment of AAs with high BP who live in low-income AA communities	CS	Mixed Methods (focus groups, environmental assessments)	Identify as AA; age ≥ 21; BP between 120 and 80 and 150 and 95 mmHg; taking <3 anti-hypertensive meds; read/speak English; BMI between 18.5 kg/m^2^ and 45.0 kg/m^2^	Post-college education; pregnant; history of diabetes, CHF, CKD, dementia, or schizophrenia; excessive alcohol use	30	Mean age 51.7 (range 34–63); 85% women; mean BMI 34.3 kg/m^2^; mean BP 136.0/84.7 mmHg; 73% 1–2 anti-hypertensive meds
Challenges to dietary hypertension self-management as described by a sample of African American older adults	Jones (2022) [23]	To document challenges with implementing DASH diet among older AAs in senior housing with HTN	CS	Qualitative (focus groups)	age ≥ 60 years; HTN; read/speak English	None listed	19	Mean age 71.6 (SD = 8.3); 87.1% women; median household income for community $27,854
Acceptability of sodium-reduced research diets, including the DASH diet, among adults with prehypertension and stage 1 hypertension	Karanja (2007) [24]	To conduct a subgroup analysis by race to examine acceptability of the DASH diet among participants in an outpatient feeding trial	RCT	Quantitative (survey/)	Age 22 years or older; SBP 120 to 159 mm Hg, DBP of 80 to 95 mm Hg	Taking BP medications; history of heart disease, renal insufficiency, hyperlipidemia, or diabetes; special diet; excessive alcohol use	354	DASH Group: 61% women; 56% AA; Mean age 47.8 years; Some college 89%; Mean BMI 28.9 and 29.5; 41% hypertension
Modifying soul food for the DASH diet plan: implications for metabolic syndrome (DASH of Soul) *	Rankins (2007) [25]	To modify soul food into DASH plan and pilot test modified DASH plan	CS	Mixed methods (focus groups, surveys)	None listed	None listed	77	Low-income AAs; 98% female; 88% AA; Mean age 50.4 (SD 11.6); 96% BMI >= 30; 36% SBP > 140; 28% DBP > 90
Mindfulness in motion and DASH in hypertensive African Americans	Wright (2021) [26]	To examine the feasibility and acceptability of a DASH intervention for community-dwelling older AAs with MCI and HTN	RCT	Mixed Methods (interviews, enrollment and attendance records)	HTN diagnosis; MCI indicated by a questionnaire	None listed	13	Mean age 74.0 (SD 5.9); 21% female; 38.5% associate degree or higher; monthly income $1996.3 (SD 1478.1); mean chronic conditions, 5 (SD 1.3); mean HTN medications, 2.1 (SD 0.9); mean SBP, 133.6 (SD 14.4); mean DBP 75.6 (SD 9.6)
Recruitment and retention of minority participants in the DASH controlled feeding trial	Vollmer (1998) [27]	To compare barriers and facilitators to DASH adherence among AA and non-Hispanic White study participants	CS	Quantitative (survey)	Age 22 years or older; DBP between 80 and 95 mm Hg; and SBP < 160 mm Hg	None listed	459 (275 AA)	Full sample: >80% some college; 66% annual household income >$30 K (DASH group only) | AA sample: 59% female; mean age 44 (SD 10); 64% 1–4 years college; 42% married; 45% income <$30 K
Effect of socioeconomic status on food availability and cost of the DASH dietary pattern * (Hand Search)	Young (2008) [28]	To examine barriers to DASH adherence among patients in high- and low-SES communities	CS	Quantitative (survey, community assessment)	Speak/Read English	None listed	100	Low-SES clinic: 74% AA
Experiences of participants of a dietary intervention trial for African Americans with hypertension and CKD	Brown (2021) [29]	To identify barriers and facilitators to DASH adherence among AAs with HTN and CKD enrolled in a trial	CS	Qualitative (interviews)	Not available	None listed	25	Mean age 62 (SD 9.3); 36% male
DASH Diet accordant foods in the homes of urban African Americans at risk for CKD	Crews (2015) [30]	To examine presence of DASH accordant foods and kitchen appliances needed to prepare DASH meals among urban AAs with uncontrolled HTN enrolled in an RCT	CS	Mixed methods (interview, home inspection	Not available	None listed	159	Mean age 57; 74% female
Barriers and facilitators to DASH diet adherence among black adults with CKD: A qualitative study	Tyson (2021) [31]	To identify barriers and facilitators to DASH adherence among AA adults with CKD with CKD stage 3 or 4	CS	Qualitative (focus groups)	Not available	None listed	22	Median age 61; 59% female; 59% CKD stage 3; 90% HTN
Self-perceived barriers and facilitators to DASH diet adherence among Black Americans with chronic kidney disease: A qualitative study	Tyson (2023) [32]	To assess DASH perceptions, its cultural compatibility, and barriers and facilitators to DASH adherence among AA adults with CKD stage 3 or 4	CS	Qualitative (focus groups, interviews)	Identify as AA; age 21 years or older; CKD diagnosis awareness; able read/understand English; no dialysis or history of kidney transplantation	None listed	22	Median age 61; 59% female; 27% married; 59% associate/bachelor degree; 27% full-time work; 39% annual household income <$25 K; 52% rarely/never have enough money to buy healthy food; 59% CKD Stage 3; 41% CKD Stage 4; 90% HTN

Notes: (*) indicates included in Spencer et al.; BP: Blood pressure; BMI: Body mass index; CKD: Chronic kidney disease; CHF: Congestive heart failure; CS: Cross sectional; DBP: Diastolic blood pressure; DASH: Dietary approaches to stop hypertension; HTN: Hypertension; MCI: Mild cognitive impairment; SES: Socioeconomic status; SD: Standard deviation; SBP: Systolic blood pressure; RCT: Randomized controlled trial.

**Table 2:** Summary of major themes and subthemes.

Major Theme	Subthemes
Availability and access of healthy foods	Poor availability of healthy foods
	Lack of transportation
	Healthy foods available/accessible
Economic cost of healthy foods	High cost of healthy foods
	High concentration of low-cost unhealthy foods
	Spoilage concerns with fresh produce
	Cost-control measures
Material resources for cooking	Inadequate cooking equipment and supplies
Food literacy	Inadequate cooking skills
	Unfamiliar cooking preparation techniques
Cultural acceptability of the DASH eating plan	Lack of culturally relevant DASH recipes (unfamiliar foods)
	Acceptability of DASH recipes and the DASH eating plan
